# Integrated Cardio–Renal–Metabolic Risk Profiling in Patients with Type 2 Diabetes: A Machine Learning-Assisted Cross-Sectional Analysis

**DOI:** 10.3390/jcm15187315

**Published:** 2026-09-20

**Authors:** Bianca-Lăcrimioara Petca, Paula-Alexandra Popovici, Andreea Diana Igna, Timea Claudia Ghitea, Mihaela Simona Popoviciu

**Affiliations:** 1 Doctoral School of Biological and Biomedical Sciences, University of Oradea, 1 University Street, 410087 Oradea, Romania; criste.biancalacrimioara@student.uoradea.ro (B.-L.P.); cipleu.paulaalexandra@student.uoradea.ro (P.-A.P.); igna.andreeadiana@student.uoradea.ro (A.D.I.); 2 Pharmacy Department, Faculty of Medicine and Pharmacy, University of Oradea, 1 University Street, 410087 Oradea, Romania; 3 Department of Preclinical Disciplines, Faculty of Medicine and Pharmacy, University of Oradea, 410028 Oradea, Romania; mihaela.popoviciu@didactic.uoradea.ro

**Keywords:** type 2 diabetes mellitus, cardiovascular risk, SCORE2-Diabetes, chronic kidney disease, cardio–renal–metabolic syndrome, machine learning, atherosclerotic cardiovascular disease, TyG index, albuminuria, risk stratification

## Abstract

**Background/Objectives:** Type 2 diabetes mellitus (T2DM) is characterized by overlapping cardiovascular, renal, metabolic, and hepatic-risk abnormalities. We characterized this integrated phenotype, examined SCORE2-Diabetes gradients, and assessed whether routinely available variables could classify established atherosclerotic cardiovascular disease (ASCVD). **Methods:** This cross-sectional study included 232 consecutive adults with T2DM. SCORE2-Diabetes tertiles in the full cohort were analyzed descriptively, and a sensitivity analysis was restricted to participants aged 40–69 years without established ASCVD or severe target-organ damage. FIB-4 was recalculated from age, aspartate aminotransferase, alanine aminotransferase, and platelet count. Elastic-net logistic regression, random forest, and gradient boosting were evaluated using nested stratified five-fold cross-validation, with all preprocessing and hyperparameter tuning confined to the training folds. **Results:** Established ASCVD was present in 49 participants (21.1%), corresponding to 4.45 events per candidate predictor. The SCORE2-Diabetes-eligible sensitivity subgroup included 118 participants (50.9%); 72.9% were in the ≥20% 10-year-risk category. FIB-4 was available for 231 participants (median 1.26 [IQR 0.96–1.80]). Nested cross-validated ROC AUCs were 0.675 (95% CI 0.582–0.763) for elastic-net logistic regression, 0.670 (0.581–0.754) for random forest, and 0.674 (0.589–0.752) for gradient boosting; balanced accuracies were 65.4%, 62.8%, and 56.2%, respectively. **Conclusions:** The cohort had a high and heterogeneous cardio–renal–metabolic burden. SCORE2-Diabetes findings from the full cohort are descriptive because the score is not intended for patients with established ASCVD or severe target-organ damage. The machine-learning models showed only modest, internally validated discrimination and are not suitable for clinical deployment without larger prospective cohorts and external validation.

## 1. Introduction

Type 2 diabetes mellitus (T2DM) is one of the leading contributors to global morbidity and mortality, affecting more than 530 million adults worldwide, with projections indicating a continuous increase over the coming decades. Cardiovascular disease remains the principal cause of death in this population, while chronic kidney disease, heart failure, and metabolic dysfunction frequently coexist and substantially worsen prognosis. Consequently, contemporary diabetes management has shifted from a glucose-centered approach toward comprehensive cardiovascular and renal risk assessment and multifactorial intervention [1,2,3].

The pathophysiological mechanisms underlying cardiovascular complications in T2DM are complex and involve the interaction of chronic hyperglycemia, insulin resistance, endothelial dysfunction, systemic inflammation, oxidative stress, dyslipidemia, and progressive renal impairment. These mechanisms promote accelerated atherosclerosis, myocardial remodeling, vascular stiffness, and microvascular injury, resulting in a markedly increased lifetime risk of myocardial infarction, stroke, heart failure, and chronic kidney disease. Importantly, these pathological processes develop simultaneously rather than independently, supporting the concept of an integrated cardio–renal–metabolic continuum [4,5,6].

Recognition of this interconnected pathophysiology has led to the development of several cardiovascular risk-prediction models specifically designed for individuals with T2DM. Among these, the recently introduced SCORE2-Diabetes algorithm incorporates traditional cardiovascular risk factors together with diabetes-specific characteristics, including age at diabetes diagnosis, glycated hemoglobin, and estimated glomerular filtration rate, to improve estimation of 10-year cardiovascular risk in individuals without established cardiovascular disease. Nevertheless, current prediction models primarily estimate future cardiovascular events and do not fully characterize the multidimensional interaction between metabolic control, renal dysfunction, systemic inflammation, hepatic fibrosis, and prevalent cardiovascular disease in routine clinical practice [7,8].

At the same time, increasing attention has been directed toward emerging biomarkers capable of reflecting different aspects of cardiometabolic dysfunction. The triglyceride–glucose (TyG) index has become an accessible surrogate marker of insulin resistance and has been associated with cardiovascular disease, non-alcoholic fatty liver disease, and diabetic complications. Albuminuria and estimated glomerular filtration rate remain the cornerstone indicators of diabetic kidney disease and independently predict cardiovascular outcomes. Likewise, inflammatory biomarkers such as C-reactive protein (CRP) and hepatic fibrosis indices, including the Fibrosis-4 (FIB-4) score, have been associated with adverse cardiometabolic outcomes, suggesting that cardiovascular risk in T2DM extends beyond conventional glycemic measurements [9,10].

Because these biomarkers reflect complementary biological pathways, their combined evaluation may provide a more comprehensive characterization of the cardio–renal–metabolic phenotype than individual parameters alone. However, the relative contribution of these routinely available clinical and laboratory variables remains incompletely understood, particularly in heterogeneous real-world populations managed in everyday clinical practice [11].

Machine-learning techniques have recently attracted considerable interest as tools capable of integrating multiple correlated clinical variables without imposing the assumptions required by traditional statistical models. Compared with conventional regression analyses, machine-learning algorithms may identify complex interactions and nonlinear relationships among cardiovascular, renal, and metabolic variables. Nevertheless, evidence regarding their incremental value in relatively small real-world cohorts of patients with T2DM remains limited, and concerns regarding overfitting, interpretability, and clinical applicability persist. Consequently, externally validated evidence supporting their routine implementation is still insufficient [12].

The present study was therefore designed to provide an integrated characterization of the cardio–renal–metabolic profile of adults with T2DM using routinely collected demographic, anthropometric, clinical, biochemical, inflammatory, renal, and hepatic-risk parameters. Specifically, we sought to (i) describe the distribution of cardiovascular, renal, and metabolic abnormalities within the study population; (ii) compare cardio–renal–metabolic characteristics across SCORE2-Diabetes risk tertiles; (iii) investigate the correlations among cardiovascular, renal, inflammatory, hepatic, and metabolic biomarkers; and (iv) evaluate the ability of machine-learning algorithms to classify participants with established atherosclerotic cardiovascular disease using routinely available clinical variables. Rather than proposing a replacement for validated cardiovascular risk scores, this study aimed to explore whether integrated cardio–renal–metabolic profiling could improve the understanding of cardiovascular-risk heterogeneity in adults with T2DM and identify variables deserving further prospective validation.

## 2. Materials and Methods

### 2.1. Study Design and Population

This cross-sectional observational study was conducted using a real-world clinical database of adults diagnosed with type 2 diabetes mellitus (T2DM) who attended a specialized outpatient diabetes and metabolic diseases clinic in Romania. The database was prospectively maintained as part of routine clinical practice and subsequently anonymized for research purposes before statistical analysis.

A total of 232 consecutive adults with confirmed T2DM were included in the present study. Eligible participants were aged 18 years or older and had complete demographic, anthropometric, clinical, cardiovascular, renal, and biochemical information required for integrated cardio–renal–metabolic assessment. Individuals with type 1 diabetes mellitus, gestational diabetes, secondary forms of diabetes, or missing essential clinical information were excluded. This study was designed and reported in accordance with the Strengthening the Reporting of Observational Studies in Epidemiology (STROBE) recommendations for cross-sectional studies.

Because this was a secondary analysis of a fixed, consecutively assembled clinical cohort, no a priori minimum sample-size calculation was performed. All eligible records available in the database were included. The 49 ASCVD cases and 11 prespecified candidate predictors corresponded to 4.45 events per candidate predictor, indicating a substantial risk of coefficient instability; consequently, the machine-learning analyses were treated as exploratory and used penalization and nested internal validation.

### 2.2. Clinical and Laboratory Assessment

Demographic characteristics, including age, sex, place of residence, diabetes duration, and smoking status, were extracted from the electronic medical records. Smoking was categorized as current, former, or never smoking and was complete for all participants. Anthropometric evaluation included body weight, height, body mass index (BMI), waist circumference, and waist-to-height ratio. Blood pressure measurements consisted of systolic and diastolic blood pressure recorded during routine clinical assessment. Baseline medication classes were also extracted, including glucose-lowering, antihypertensive, antiplatelet/antithrombotic, and lipid-lowering therapies; drug dose, duration, treatment changes, and adherence were not available.

Clinical cardiovascular history included physician-documented hypertension, dyslipidemia, myocardial infarction, coronary revascularization, stroke, peripheral arterial disease, heart failure, and atrial fibrillation. Established atherosclerotic cardiovascular disease (ASCVD) was defined as the presence of at least one documented history of myocardial infarction, coronary revascularization, ischemic stroke, or peripheral arterial disease.

Laboratory evaluation comprised fasting plasma glucose, glycated hemoglobin (HbA1c), total cholesterol, low-density lipoprotein cholesterol, high-density lipoprotein cholesterol, triglycerides, serum creatinine, serum urea, estimated glomerular filtration rate (eGFR), urinary albumin-to-creatinine ratio (UACR), C-reactive protein (CRP), aspartate aminotransferase (AST; U/L), alanine aminotransferase (ALT; U/L), and platelet count (10^3^/µL). The triglyceride–glucose (TyG) index was calculated as ln[fasting triglycerides (mg/dL) × fasting glucose (mg/dL)/2]. FIB-4 was calculated as age (years) × AST (U/L)/[platelet count (10^3^/µL) × √ALT (U/L)]. One platelet value of 0 was considered biologically impossible and was treated as missing, leaving 231 valid FIB-4 values. The recalculated FIB-4 values agreed with the values stored in the database. SCORE2-Diabetes values recorded in the database had been calculated using the ESC variables, including current smoking status; the completeness and coding of smoking status were rechecked for this revision.

### 2.3. Definitions

Body mass index was classified according to the World Health Organization criteria into normal weight (18.5–24.9 kg/m^2^), overweight (25.0–29.9 kg/m^2^), class I obesity (30.0–34.9 kg/m^2^), class II obesity (35.0–39.9 kg/m^2^), and class III obesity (≥40.0 kg/m^2^). Reduced renal function was defined as an estimated glomerular filtration rate below 60 mL/min/1.73 m^2^, whereas albuminuria was defined as a urinary albumin-to-creatinine ratio of at least 30 mg/g. Poor glycemic control was considered when HbA1c was ≥7.0%, while increased insulin resistance was defined as a TyG index greater than 9.04. Hypertension, dyslipidemia, heart failure, atrial fibrillation, myocardial infarction, stroke, and peripheral arterial disease were recorded according to physician-established diagnoses documented in the medical records.

The full-cohort SCORE2-Diabetes tertile analysis was prespecified as descriptive. A sensitivity analysis followed the formal intended-use population: age 40–69 years, no established ASCVD, and no severe target-organ damage. Severe target-organ damage was operationalized from available variables as eGFR <45 mL/min/1.73 m^2^ irrespective of albuminuria, eGFR 45–59 mL/min/1.73 m^2^ with UACR ≥ 30 mg/g, and UACR > 300 mg/g; or microvascular disease at three sites (albuminuria, diabetic retinopathy, and peripheral neuropathy). Within the eligible subgroup, guideline SCORE2-Diabetes categories were <5%, 5–<10%, 10–<20%, and ≥20%.

### 2.4. Study Objectives

The primary objective of this study was to characterize the integrated cardio–renal–metabolic profile of adults with T2DM using routinely available demographic, anthropometric, cardiovascular, renal, inflammatory, hepatic, and metabolic variables. Secondary objectives included comparison of clinical characteristics across SCORE2-Diabetes risk tertiles; investigation of the relationships among cardiovascular, renal, inflammatory, hepatic, and metabolic biomarkers; and evaluation of machine-learning algorithms for the classification of established ASCVD. Because of the cross-sectional study design, the analyses were intended to describe associations rather than establish causal relationships.

### 2.5. Statistical Analysis

Continuous variables were evaluated for normality using the Shapiro–Wilk test together with visual inspection of histograms and quantile–quantile plots. Variables with normal distributions are presented as mean ± standard deviation, whereas skewed variables are reported as median and interquartile range. Categorical variables are expressed as absolute frequencies and percentages.

Comparisons among the observed SCORE2-Diabetes tertiles in the full cohort were performed using one-way analysis of variance for normally distributed variables, or the Kruskal–Wallis test for non-normally distributed variables. Whenever significant overall differences were identified, appropriate post hoc analyses with correction for multiple comparisons were performed. Categorical variables were compared using Pearson’s chi-square test or Fisher’s exact test whenever expected cell counts were small. The sensitivity analysis repeated the principal SCORE2-Diabetes summaries and correlations only in formally eligible participants.

Relationships among continuous variables were assessed using Spearman’s rank correlation coefficient because several metabolic and renal biomarkers demonstrated non-normal distributions. To reduce the likelihood of false-positive findings arising from multiple testing, *p*-values from both the full-cohort and eligibility-restricted correlation analyses were adjusted using the Benjamini–Hochberg false discovery-rate procedure. Statistical significance was defined as a two-sided *p*-value below 0.05.

### 2.6. Machine-Learning Analysis

Machine-learning analyses were performed to investigate whether routinely available cardio–renal–metabolic variables could classify participants with established ASCVD. To prevent target leakage, SCORE2-Diabetes and the cardiovascular diagnoses defining the composite ASCVD outcome were excluded from the predictor set. The analysis was explicitly exploratory because only 49 ASCVD events were available.

Eleven predictors were prespecified on clinical and domain-coverage grounds before model fitting: age, sex, diabetes duration, body mass index, systolic blood pressure, HbA1c, TyG index, eGFR, UACR, CRP, and FIB-4. One clinically implausible HbA1c value (114%) was treated as missing. Median imputation and, when applicable, standardization were performed inside each training fold to prevent information leakage. Because smoking data were available, an additional sensitivity model added current smoking to the penalized logistic-regression predictor set.

Three supervised algorithms were evaluated: elastic-net penalized logistic regression, random forest, and gradient boosting. Performance was estimated using nested stratified five-fold cross-validation with a fixed random seed (20260910). In every outer training set, four-fold inner cross-validation tuned elastic-net C (0.03, 0.1, 0.3, 1, 3, or 10) and l1 ratio (0, 0.25, 0.5, 0.75, or 1); random-forest maximum depth (2, 4, or unrestricted), minimum leaf size (2, 5, or 10), and maximum features (square root or 0.5); and gradient-boosting estimators (50, 100, or 200), learning rate (0.02, 0.05, or 0.1), maximum depth (1, 2, or 3), and minimum leaf size (5, 10, or 20). ROC AUC was the tuning criterion. Each outer-test observation was predicted only by a model that had not used that observation for preprocessing, tuning, threshold selection, or fitting. Sensitivity, specificity, and balanced accuracy used a Youden threshold derived solely from inner out-of-fold predictions in the corresponding outer training set. ROC AUC, average precision, Brier score, sensitivity, specificity, and balanced accuracy were reported; 95% confidence intervals were obtained from 3000 subject-level bootstrap resamples of outer out-of-fold predictions. Coefficients from a final full-cohort penalized model were reported only descriptively and not as independently validated causal effects.

### 2.7. Ethical Considerations

This study was conducted in accordance with the ethical principles of the Declaration of Helsinki. All clinical data were anonymized before statistical analysis, and no personally identifiable information was available to the investigators. This study’s protocol was approved by the Ethical Committee of the Faculty of Medicine and Pharmacy, University of Oradea (Approval No. 42, 31 October 2025). Informed consent was obtained from all subjects involved in this study.

## 3. Results

### 3.1. Baseline Clinical and Cardio–Renal–Metabolic Characteristics

This study included 232 adults with type 2 diabetes mellitus, of whom 122 (52.6%) were women and 110 (47.4%) were men. The mean age was 62.58 ± 10.10 years, with a median of 64 years (IQR 55–70 years). Current smoking was recorded in 29 participants (12.5%), former smoking in 40 (17.2%), and never smoking in 163 (70.3%). A nearly balanced residential distribution was observed, with 119 participants (51.3%) living in urban areas and 113 (48.7%) living in rural areas. The median diabetes duration was 6 years (IQR 2–14 years), and the mean duration was 8.31 ± 7.65 years.

The cohort was characterized by a substantial burden of excess adiposity. Mean BMI was 31.21 ± 5.70 kg/m^2^, and median BMI was 30.40 kg/m^2^ (IQR 27.11–34.13 kg/m^2^). Overall, 79 participants (34.1%) were overweight, and 127 (54.7%) had obesity. Mean waist circumference was 106.13 ± 13.99 cm. Mean waist-to-height ratio was 0.636 ± 0.086, and 225 participants (97.0%) had a value > 0.5, indicating very limited contrast for this anthropometric risk marker within the cohort.

Hypertension was present in 208 participants (89.7%), and dyslipidemia was identified in 219 (94.4%). The mean systolic and diastolic blood pressures were 145.87 ± 20.48 mmHg and 86.69 ± 11.21 mmHg, respectively. Regarding established cardiovascular disease, 23 participants (9.9%) had a history of myocardial infarction, 14 (6.0%) had experienced a stroke, and 25 (10.8%) had peripheral arterial disease. Heart failure was recorded in 61 participants (26.3%), while atrial fibrillation was present in 18 (7.8%). A composite history of atherosclerotic cardiovascular disease, defined by myocardial infarction, coronary revascularization, stroke, or peripheral arterial disease, was identified in 49 participants (21.1%).

Glycemic control was heterogeneous. The median fasting plasma glucose concentration was 143.5 mg/dL (IQR: 118.0–188.5 mg/dL), and the mean was 163.43 ± 72.25 mg/dL. After exclusion of one clinically implausible value, HbA1c was available for 231 participants. The mean HbA1c was 7.69 ± 2.00%, with a median of 7.00% (IQR: 6.25–8.60%).

Renal function also varied considerably across the cohort. The mean serum creatinine concentration was 0.88 ± 0.32 mg/dL, while the median estimated glomerular filtration rate was 82.97 mL/min/1.73 m^2^ (IQR: 59.91–94.89 mL/min/1.73 m^2^). An estimated glomerular filtration rate of at least 90 mL/min/1.73 m^2^ was observed in 85 participants (36.6%), whereas 88 (37.9%) had values between 60 and 89 mL/min/1.73 m^2^. Moderate renal impairment, defined by an estimated glomerular filtration rate between 30 and 59 mL/min/1.73 m^2^, was present in 55 participants (23.7%), and four participants (1.7%) had values below 30 mL/min/1.73 m^2^. An albumin-to-creatinine ratio of at least 30 mg/g was identified in 80 participants (34.5%).

The median CRP concentration was 3.50 mg/L (IQR 1.50–6.93 mg/L), and the median TyG index was 9.15 (IQR 8.79–9.71). AST and ALT medians were 23 U/L (IQR 18–29) and 22 U/L (IQR 15–32), respectively; the valid platelet-count median was 239 × 10^3^/µL (IQR 194–285). FIB-4 was available for 231 participants and had a median of 1.26 (IQR 0.96–1.80): 146 (63.2%) had FIB-4 < 1.45, 72 (31.2%) had values of 1.45–3.25, and 13 (5.6%) had values >3.25. The mean recorded SCORE2-Diabetes value in the full cohort was 34.87 ± 15.71%, with a median of 34.25% (IQR 22.98–45.75%); this full-cohort estimate is reported descriptively because not all participants met formal SCORE2-Diabetes eligibility criteria (Table 1).

### 3.2. Biochemical and Cardio–Renal–Metabolic Profile

The biochemical assessment demonstrated substantial metabolic heterogeneity within the study population. The median fasting plasma glucose concentration was 143.5 mg/dL (IQR: 118.0–188.5 mg/dL), while HbA1c measurements, available for 231 participants after exclusion of one clinically implausible value, showed a median of 7.00% (IQR: 6.25–8.60%), indicating that a considerable proportion of patients did not achieve recommended glycemic targets.

Markers of insulin resistance also suggested an unfavorable metabolic profile. The median triglyceride–glucose (TyG) index was 9.15 (IQR: 8.79–9.71), and 137 participants (59.1%) presented values above the predefined threshold of 9.04, consistent with increased insulin resistance and elevated cardiometabolic risk.

Renal function showed marked interindividual variability. The median serum creatinine concentration was 0.83 mg/dL (IQR: 0.66–1.00 mg/dL), while the median estimated glomerular filtration rate (eGFR) was 82.97 mL/min/1.73 m^2^ (IQR: 59.91–94.89 mL/min/1.73 m^2^). Overall, 59 patients (25.4%) had an eGFR below 60 mL/min/1.73 m^2^, indicating at least moderate chronic kidney disease. Furthermore, urinary albumin excretion was increased in a substantial proportion of participants, with 80 individuals (34.5%) presenting an albumin-to-creatinine ratio ≥30 mg/g, compatible with diabetic kidney involvement.

Systemic inflammation was generally low to moderate but highly variable across the cohort. Median C-reactive protein concentration was 3.50 mg/L (IQR: 1.50–6.93 mg/L), reflecting the chronic low-grade inflammatory state frequently associated with obesity, insulin resistance, and type 2 diabetes.

**Figure 1:**
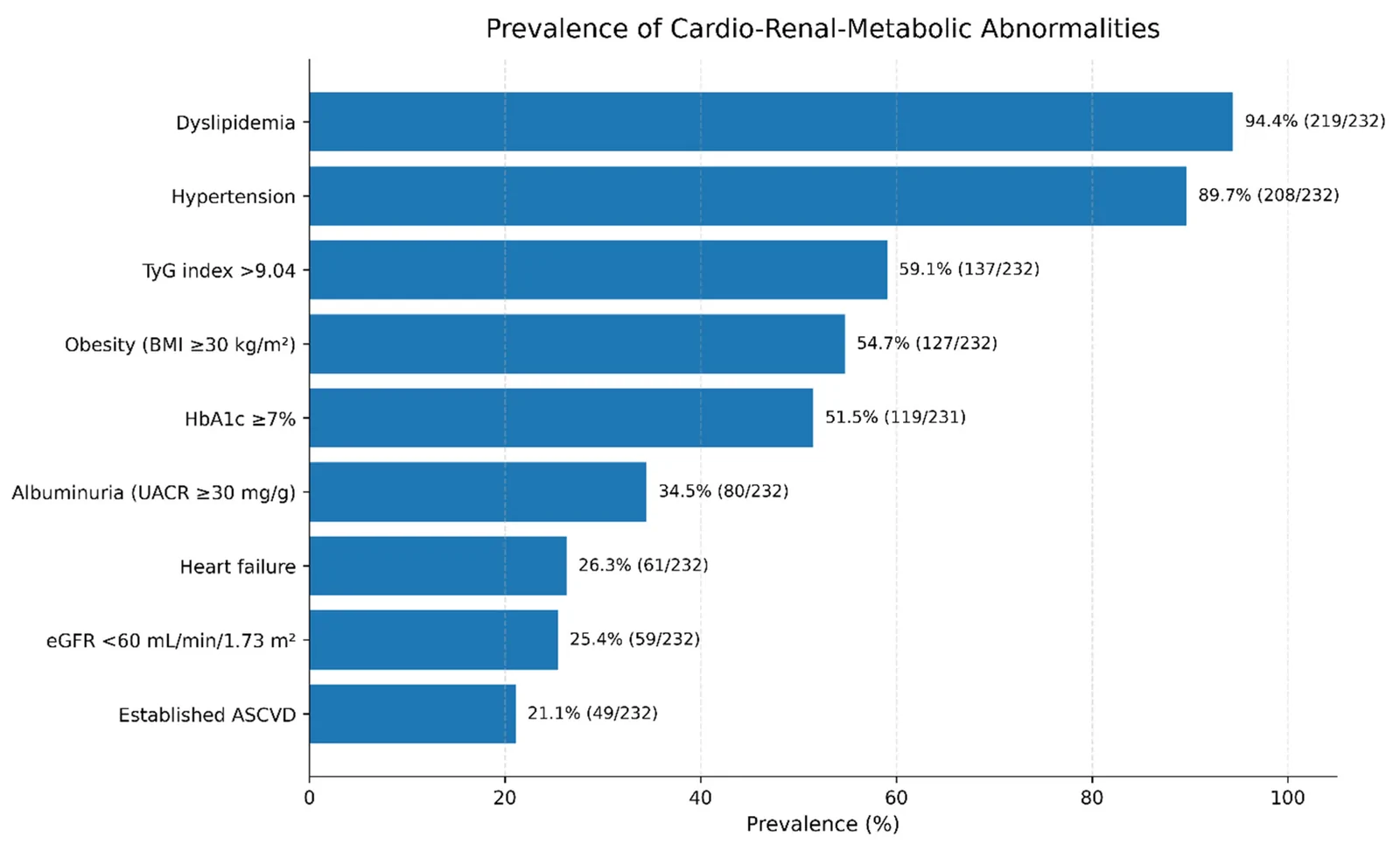
Prevalence of cardio–renal–metabolic abnormalities in the study cohort. Horizontal bars show the prevalence of major cardio–renal–metabolic abnormalities in the study cohort. Dyslipidemia and hypertension were the most frequent conditions, affecting 94.4% and 89.7% of participants, respectively. Elevated TyG index, obesity, and HbA1c ≥ 7% were present in more than half of the cohort. Values are presented as percentages and absolute counts. HbA1c was available for 231 participants after one clinically implausible value was treated as missing; all other indicators were available for 232 participants. Established ASCVD was defined as a history of myocardial infarction, coronary revascularization, stroke, or peripheral arterial disease.

The estimated 10-year cardiovascular risk was considerable. The mean SCORE2-Diabetes value was 34.87 ± 15.71%, highlighting the overall high cardiovascular burden of the cohort and supporting the rationale for developing an integrated cardio–renal–metabolic risk model incorporating metabolic, renal, inflammatory, and cardiovascular variables (Table 2 and Figure 1).

Baseline treatment classes were available. Metformin/biguanide therapy was recorded in 123 participants (53.0%), an SGLT2 inhibitor in 104 (44.8%), a GLP-1 receptor agonist in 36 (15.5%), a DPP-4 inhibitor in 34 (14.7%), a sulfonylurea in 19 (8.2%), basal insulin in 73 (31.5%), and rapid-acting insulin in 22 (9.5%). Cardiovascular therapies included beta-blockers in 125 (53.9%), diuretics in 115 (49.6%), ACE inhibitors in 111 (47.8%), angiotensin-receptor blockers in 59 (25.4%), antiplatelet/antithrombotic therapy in 75 (32.3%), statins in 113 (48.7%), and ezetimibe in 28 (12.1%). Because therapies overlapped, percentages do not sum to 100%. These data describe prescriptions at the index assessment and do not support treatment-effect inference.

### 3.3. Clinical and Metabolic Characteristics According to SCORE2-Diabetes Risk Tertiles

Participants were stratified into tertiles according to the observed distribution of the SCORE2-Diabetes estimated 10-year cardiovascular risk. The low-risk tertile included 78 participants with SCORE2-Diabetes values ≤26.1%, the intermediate-risk tertile included 76 participants with values >26.1% and <42.2%, and the high-risk tertile included 78 participants with values ≥42.2%.

Age increased substantially across the three risk categories, from 53.99 ± 6.34 years in the first tertile to 63.64 ± 8.10 years in the second tertile and 70.14 ± 8.26 years in the third tertile (*p* < 0.001). Diabetes duration also increased progressively, with median values of 2.5 years (IQR: 1.0–6.0), 8.0 years (IQR: 3.75–15.0), and 10.0 years (IQR: 4.0–19.0), respectively (*p* < 0.001).

Systolic blood pressure was significantly higher among participants in the highest-risk tertile, increasing from 138.81 ± 19.31 mmHg in T1 to 144.09 ± 16.80 mmHg in T2 and 154.67 ± 21.88 mmHg in T3 (*p* < 0.001). In contrast, body mass index, waist circumference, and diastolic blood pressure did not differ significantly among the three groups.

Glycemic status became progressively less favorable across increasing cardiovascular-risk categories. Median fasting plasma glucose increased from 129.0 mg/dL (IQR: 107.75–157.0) in T1 to 146.5 mg/dL (IQR: 123.5–190.25) in T2 and 167.5 mg/dL (IQR: 123.5–225.75) in T3 (*p* < 0.001). Similarly, median HbA1c increased from 6.40% (IQR: 6.00–7.07%) to 7.20% (IQR: 6.30–8.53%) and 7.90% (IQR: 6.90–10.30%), respectively (*p* < 0.001). The TyG index also differed among the groups; however, the magnitude of the difference was modest, with median values of 9.02, 9.14, and 9.31 in T1, T2, and T3, respectively (*p* = 0.039).

Renal impairment was more pronounced in the highest-risk category. Median serum creatinine increased from 0.78 mg/dL (IQR: 0.65–0.88) in T1 to 0.83 mg/dL (IQR: 0.66–1.01) in T2 and 0.92 mg/dL (IQR: 0.68–1.12) in T3 (*p* = 0.003). In parallel, median estimated glomerular filtration rate decreased from 94.15 mL/min/1.73 m^2^ (IQR: 81.25–101.34) to 80.43 mL/min/1.73 m^2^ (IQR: 60.20–92.25) and 64.41 mL/min/1.73 m^2^ (IQR: 47.83–87.20), respectively (*p* < 0.001). Urinary albumin-to-creatinine ratio did not differ significantly across the risk tertiles (*p* = 0.132).

The proportion of women was higher in the third tertile than in the first two tertiles (69.2% versus 43.6% and 44.7%, respectively; *p* = 0.001). Hypertension prevalence increased from 80.8% in T1 to 88.2% in T2 and 100% in T3 (*p* < 0.001). Established atherosclerotic cardiovascular disease was identified in 15.4%, 17.1%, and 30.8% of participants, respectively (*p* = 0.036), while heart-failure prevalence increased from 11.5% in T1 to 30.3% in T2 and 37.2% in T3 (*p* < 0.001). Dyslipidemia was highly prevalent in all groups and showed only a borderline between-group difference (*p* = 0.051).

**Figure 2:**
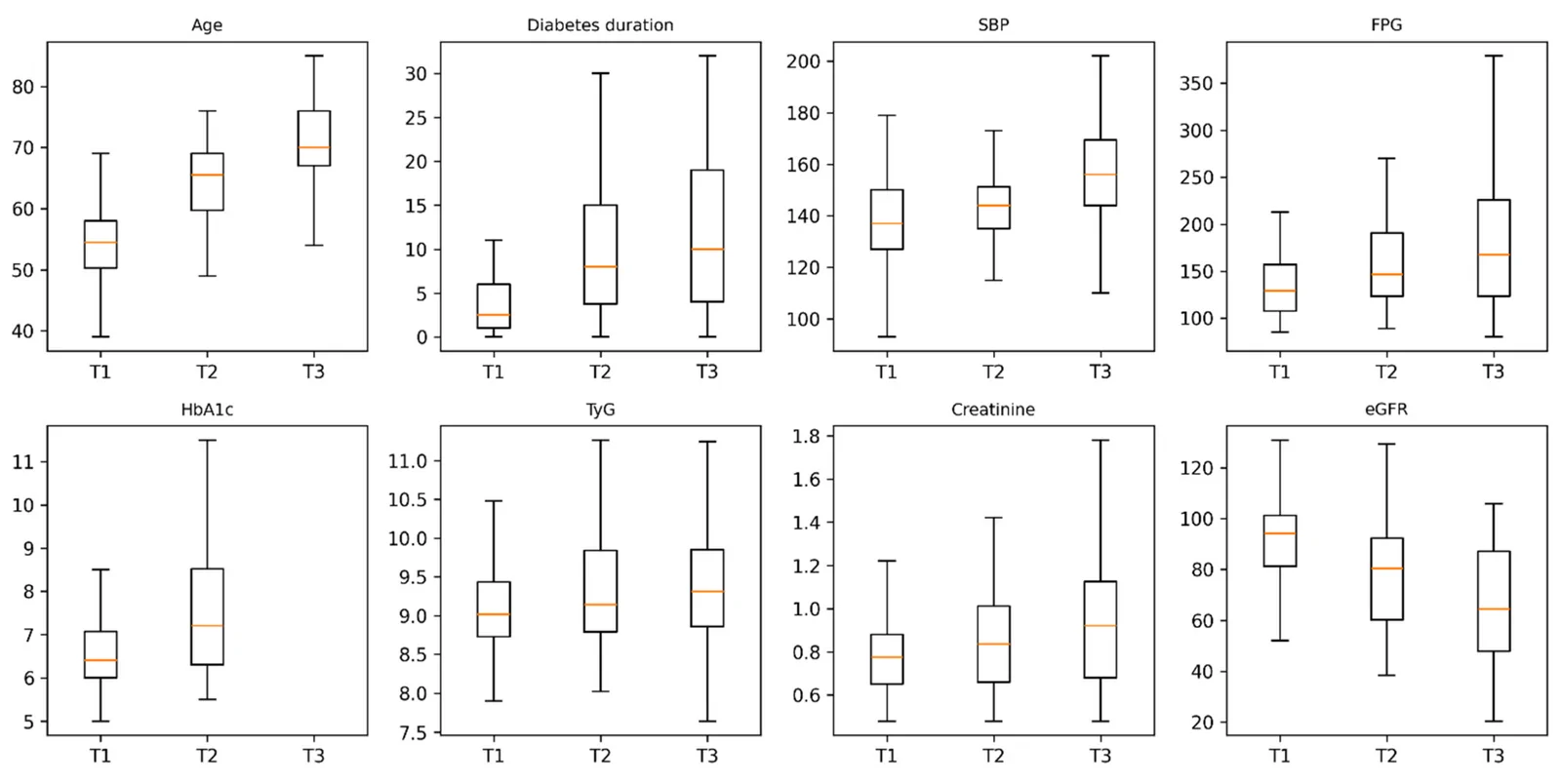
Distribution of key cardio–renal–metabolic parameters across SCORE2-Diabetes risk tertiles. Boxplots illustrate the distribution of age, diabetes duration, systolic blood pressure, fasting plasma glucose, HbA1c, TyG index, serum creatinine, and estimated glomerular filtration rate across the three SCORE2-Diabetes risk tertiles. T1 included participants with SCORE2-Diabetes ≤26.1%, T2 included those with values >26.1% and <42.2%, and T3 included those with values ≥42.2%. Between-group differences were assessed using one-way analysis of variance or the Kruskal–Wallis test, as appropriate. Horizontal lines indicate medians, boxes represent interquartile ranges, and whiskers denote values within 1.5 times the interquartile range.

C-reactive protein concentrations tended to increase across cardiovascular-risk tertiles, from a median of 2.90 mg/L in T1 to 3.70 mg/L in T2 and 4.60 mg/L in T3; however, this difference did not reach statistical significance (*p* = 0.086). Overall, increasing SCORE2-Diabetes risk was accompanied by a longer duration of diabetes, poorer glycemic control, higher systolic blood pressure, declining renal function, and a greater burden of cardiovascular comorbidities (Table 3 and Figure 2).

A sensitivity analysis restricted SCORE2-Diabetes to the formal intended-use population. Of 232 participants, 118 (50.9%) were aged 40–69 years and had neither established ASCVD nor severe target-organ damage according to the available renal and microvascular variables. In this subgroup, the mean SCORE2-Diabetes estimate was 26.66 ± 11.51%, and the median was 25.30% (IQR 18.43–34.03%). No participant had a calculated risk <5%; seven (5.9%) were in the 5–<10% category, 25 (21.2%) in the 10–<20% category, and 86 (72.9%) in the ≥20% category. After false-discovery-rate correction, the score remained associated with age (ρ = 0.718), HbA1c (ρ = 0.450), diabetes duration (ρ = 0.354), systolic blood pressure (ρ = 0.252), eGFR (ρ = −0.262), FIB-4 (ρ = 0.214), and TyG (ρ = 0.202), but not with BMI (ρ = −0.046), waist circumference (ρ = 0.077), or UACR (ρ = 0.010). These associations remain partly structural because several variables are components of the score.

### 3.4. Correlation Analysis of Cardiovascular, Renal, and Metabolic Parameters

Spearman correlation analysis was performed to examine the relationships between SCORE2-Diabetes and the principal demographic, clinical, metabolic, renal, inflammatory, and hepatic-risk variables. To reduce the probability of false-positive findings resulting from multiple comparisons, *p*-values were adjusted using the Benjamini–Hochberg false-discovery-rate procedure.

SCORE2-Diabetes showed its strongest positive correlation with age (ρ = 0.736, adjusted *p* < 0.001). Moderate positive correlations were also observed with HbA1c (ρ = 0.424, adjusted *p* < 0.001), diabetes duration (ρ = 0.389, adjusted *p* < 0.001), systolic blood pressure (ρ = 0.359, adjusted *p* < 0.001), serum urea (ρ = 0.354, adjusted *p* < 0.001), and FIB-4 (ρ = 0.323, adjusted *p* < 0.001).

A moderate inverse correlation was identified between SCORE2-Diabetes and estimated glomerular filtration rate (ρ = −0.446, adjusted *p* < 0.001), indicating that increasing estimated cardiovascular risk was associated with declining renal function. Serum creatinine was positively correlated with SCORE2-Diabetes (ρ = 0.223, adjusted *p* = 0.001), further supporting the relationship between cardiovascular-risk burden and renal impairment.

Glycemic parameters demonstrated significant positive associations with cardiovascular risk. Fasting plasma glucose correlated positively with SCORE2-Diabetes (ρ = 0.289, adjusted *p* < 0.001), while the TyG index showed a weaker but statistically significant correlation (ρ = 0.171, adjusted *p* = 0.017). In contrast, total cholesterol, LDL cholesterol, HDL cholesterol, and triglyceride concentrations were not individually associated with SCORE2-Diabetes after correction for multiple comparisons.

No significant adjusted correlations were observed between SCORE2-Diabetes and body mass index, waist circumference, diastolic blood pressure, urinary albumin-to-creatinine ratio, or C-reactive protein. Although the urinary albumin-to-creatinine ratio (ρ = 0.130, unadjusted *p* = 0.048) and C-reactive protein (ρ = 0.139, unadjusted *p* = 0.035) initially showed weak positive associations, these relationships did not remain statistically significant after false-discovery-rate correction.

Additional analyses demonstrated strong internal relationships among the metabolic and renal biomarkers. HbA1c was strongly correlated with fasting plasma glucose (ρ = 0.677, *p* < 0.001). The TyG index was strongly correlated with triglyceride concentrations (ρ = 0.896, *p* < 0.001) and moderately correlated with fasting plasma glucose (ρ = 0.541, *p* < 0.001), consistent with its mathematical derivation from these two variables.

Estimated glomerular filtration rate was strongly and inversely correlated with serum creatinine (ρ = −0.891, *p* < 0.001). Urinary albumin-to-creatinine ratio was positively associated with HbA1c (ρ = 0.258, *p* < 0.001) and systolic blood pressure (ρ = 0.157, *p* = 0.017), suggesting that poorer glycemic control and higher systolic pressure were associated with greater renal albumin loss. C-reactive protein was weakly correlated with body mass index (ρ = 0.186, *p* = 0.004) and waist circumference (ρ = 0.196, *p* = 0.003), supporting a relationship between adiposity and low-grade systemic inflammation.

FIB-4 was inversely correlated with estimated glomerular filtration rate (ρ = −0.268, *p* < 0.001) and positively correlated with age (ρ = 0.519, *p* < 0.001). The latter relationship should be interpreted cautiously because age is included directly in the FIB-4 calculation.

**Figure 3:**
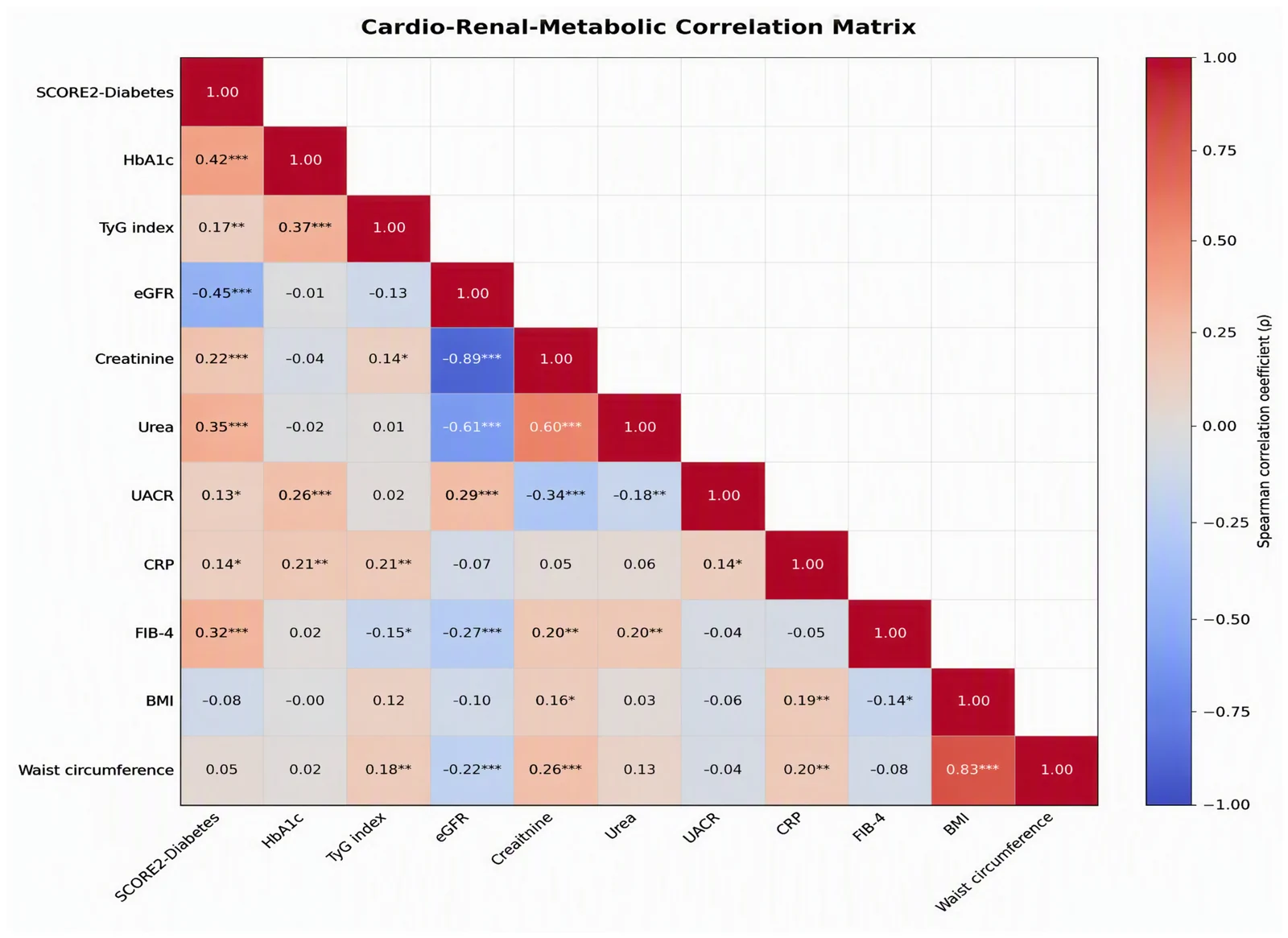
Spearman correlation matrix of cardio–renal–metabolic parameters in patients with type 2 diabetes. The heatmap presents pairwise Spearman correlation coefficients among SCORE2-Diabetes, glycemic, renal, inflammatory, hepatic-risk, and anthropometric parameters. Positive correlations are shown in red and inverse correlations in blue. Values within cells represent Spearman’s ρ coefficients. Statistical significance is indicated as follows: * *p* < 0.05, ** *p* < 0.01, and *** *p* < 0.001. HbA1c was available for 231 participants after one clinically implausible value was treated as missing; all other variables were available for 232 participants.

Overall, the correlation pattern indicated that elevated cardiovascular risk was most strongly associated with older age, poorer glycemic control, longer diabetes duration, higher systolic blood pressure, impaired renal function, and increased FIB-4 values. However, correlations involving age, blood pressure, glycemic status, and renal function should not be interpreted as external validation of SCORE2-Diabetes because several of these variables are incorporated directly or indirectly into its calculation (Table 4 and Figure 3).

### 3.5. Nested Cross-Validated Machine-Learning Classification of Established Atherosclerotic Cardiovascular Disease

Machine-learning analyses were performed to investigate whether the integrated cardio–renal–metabolic profile could discriminate between participants with and without established atherosclerotic cardiovascular disease. Established ASCVD was defined as a documented history of myocardial infarction, coronary revascularization, stroke, or peripheral arterial disease. Overall, 49 participants (21.1%) met the composite ASCVD definition, whereas 183 (78.9%) had no recorded ASCVD.

To minimize target leakage and mathematical redundancy, SCORE2-Diabetes and the individual diagnoses used to define the ASCVD outcome were excluded from the predictor set. Eleven clinically relevant candidate variables were retained: age, sex, diabetes duration, body mass index, systolic blood pressure, HbA1c, TyG index, estimated glomerular filtration rate, urinary albumin-to-creatinine ratio, C-reactive protein, and FIB-4. One clinically implausible HbA1c value was treated as missing. Missing predictor values were imputed using the median calculated within the modelling pipeline, and continuous variables were standardized before model fitting.

All three algorithms were tuned in the inner folds of a nested stratified five-fold procedure and evaluated using outer out-of-fold predictions. Because the outcome prevalence was 21.1%, average precision was interpreted relative to this prevalence. Threshold-dependent metrics were calculated using thresholds selected only within the corresponding outer training data.

Elastic-net logistic regression achieved an outer out-of-fold ROC AUC of 0.675 (95% bootstrap CI 0.582–0.763), average precision of 0.368 (0.265–0.523), and Brier score of 0.156 (0.127–0.186). Training-derived thresholds yielded 67.3% sensitivity, 63.4% specificity, and 65.4% balanced accuracy.

Random forest achieved a ROC AUC of 0.670 (95% CI 0.581–0.754), average precision of 0.395 (0.272–0.534), Brier score of 0.156 (0.128–0.186), sensitivity of 55.1%, specificity of 70.5%, and balanced accuracy of 62.8%. Gradient boosting achieved a ROC AUC of 0.674 (0.589–0.752), average precision of 0.402 (0.281–0.535), Brier score of 0.156 (0.127–0.187), sensitivity of 40.8%, specificity of 71.6%, and balanced accuracy of 56.2%. The confidence intervals overlapped substantially.

When the penalized logistic model was tuned on the complete dataset for descriptive coefficient reporting, the selected l1 ratio was 0 (the ridge boundary of the elastic-net grid) with C = 0.03. All coefficients were therefore retained but strongly shrunk. The largest standardized coefficients were observed for UACR, diabetes duration, age, eGFR, HbA1c, and FIB-4; coefficient signs and magnitudes should not be interpreted causally.

The standardized coefficients were UACR β = 0.227, diabetes duration β = 0.183, age β = 0.179, eGFR β = −0.177, HbA1c β = 0.126, and FIB-4 β = 0.123; the remaining absolute coefficients were <0.10. Adding current smoking to the penalized logistic sensitivity model produced a ROC AUC of 0.690 (95% CI 0.600–0.776), but the confidence interval overlapped that of the prespecified model and balanced accuracy was 59.3%, providing no clear evidence of clinically meaningful incremental performance.

Overall, the three nested cross-validated models demonstrated similarly modest discrimination, and the wide confidence intervals reflected the limited number of ASCVD events. The results support the use of machine learning only as exploratory profiling in this cohort, not as a clinically deployable classifier (Table 5 and Table 6).

**Figure 4:**
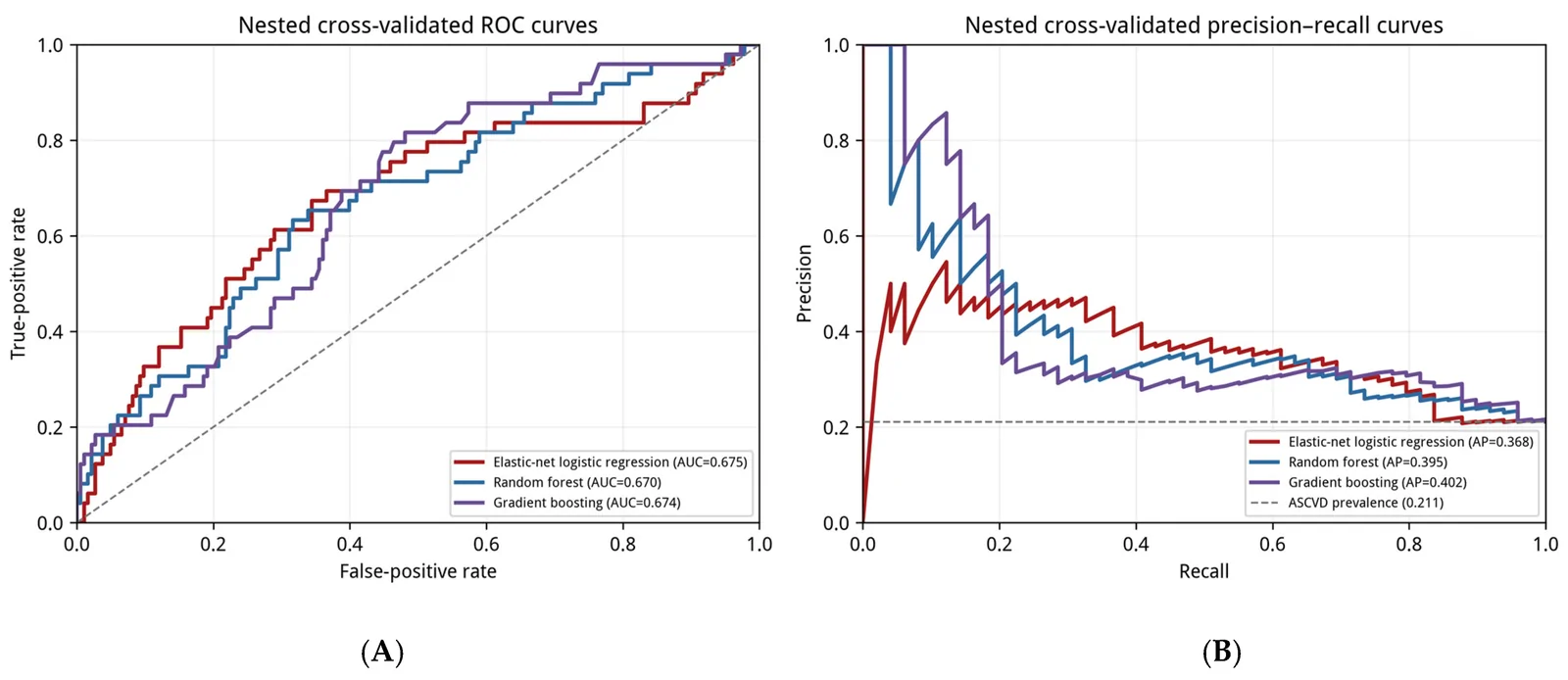
Nested cross-validated performance of machine-learning models for the classification of established ASCVD. (**A**) Receiver-operating-characteristic curves based exclusively on outer out-of-fold predictions. (**B**) Precision–recall curves; the dashed horizontal line indicates the ASCVD prevalence (21.1%). All preprocessing and hyperparameter tuning were confined to training folds.

Figure 4 presents the nested cross-validated discrimination of the three machine-learning models.

## 4. Discussion

The present study provides an integrated characterization of the cardiovascular, renal, metabolic, and inflammatory burden in a real-world cohort of adults with type 2 diabetes mellitus. The principal findings were the high prevalence of hypertension, dyslipidemia, obesity, insulin-resistance-related metabolic abnormalities, and renal involvement; the progressive deterioration of glycemic and renal parameters across increasing SCORE2-Diabetes tertiles; and the modest ability of machine-learning models to classify participants with established atherosclerotic cardiovascular disease. Collectively, the findings emphasize the marked heterogeneity of risk among patients with type 2 diabetes and the need to consider cardiovascular, renal, and metabolic abnormalities simultaneously rather than evaluating glycemic control in isolation.

### 4.1. High Cardio–Renal–Metabolic Burden in Type 2 Diabetes

The study population was characterized by a substantial accumulation of conventional and diabetes-specific risk factors. Hypertension was present in 89.7% of participants, dyslipidemia in 94.4%, and obesity in 54.7%. Furthermore, 59.1% of the cohort had a TyG index above 9.04, and more than half of the participants with available measurements had an HbA1c value of at least 7%. These findings indicate that a large proportion of the cohort had persistent metabolic risk despite being under clinical care.

The observed clustering of obesity, hypertension, dyslipidemia, and suboptimal glycemic control is clinically important because cardiovascular risk in type 2 diabetes is determined by the cumulative interaction of multiple risk pathways. Current European recommendations therefore prioritize comprehensive cardiovascular-risk assessment and multifactorial management rather than a strategy focused exclusively on glucose reduction [13,14,15].

Although the median TyG index increased significantly across the SCORE2-Diabetes tertiles, the magnitude of the difference was relatively small, and its direct correlation with SCORE2-Diabetes was weak. This suggests that TyG captures a metabolic dimension that is related to, but not fully represented by, conventional cardiovascular-risk estimation. However, because TyG is mathematically derived from fasting glucose and triglyceride concentrations, its predictive contribution should be assessed alongside, rather than simultaneously with, its component variables to avoid redundancy and multicollinearity [16,17].

The absence of significant differences in BMI, waist circumference, and waist-to-height ratio across SCORE2-Diabetes tertiles requires cautious interpretation. Excess adiposity was nearly ubiquitous: 54.7% had obesity and 97.0% had a waist-to-height ratio >0.5. This restricted between-person contrast can create a ceiling effect and reduce discriminatory power even when adiposity is clinically important. BMI does not distinguish fat distribution, visceral or ectopic adiposity, or lean mass, whereas waist circumference can add risk information beyond BMI but remains sensitive to measurement technique and sex- and ethnicity-specific thresholds [18]. BMI and waist circumference have nevertheless predicted elevated SCORE2/SCORE2-OP risk in other populations, with performance differing by sex [19]. Recent longitudinal evidence in T2DM further suggests that waist-to-height ratio is associated with cardiovascular outcomes and mortality, including among individuals without conventionally defined abdominal obesity [20]. Thus, the present null differences should not be interpreted as evidence that anthropometry is irrelevant; rather, traditional measures may be insufficiently granular in a cohort with uniformly high metabolic burden.

### 4.2. Renal Involvement as a Central Component of Cardiovascular Risk

Renal abnormalities were frequent in the analyzed cohort. Approximately one-quarter of participants had an eGFR below 60 mL/min/1.73 m^2^, while 34.5% had a urinary albumin-to-creatinine ratio of at least 30 mg/g. These proportions are compatible with the recognized high burden of chronic kidney disease among people with diabetes. Current diabetes-care standards indicate that diabetic kidney disease affects approximately 20–40% of individuals with diabetes and emphasize that both reduced eGFR and increased albuminuria are independently associated with cardiovascular and renal outcomes [21,22].

A clear decline in renal function was observed across increasing SCORE2-Diabetes tertiles. Median eGFR decreased from 94.15 mL/min/1.73 m^2^ in the lowest tertile to 64.41 mL/min/1.73 m^2^ in the highest tertile, while serum creatinine increased progressively. SCORE2-Diabetes was also inversely correlated with eGFR and positively correlated with serum creatinine and urea. These relationships reinforce the close clinical interaction between renal impairment and cardiovascular-risk accumulation in type 2 diabetes [23,24].

Nevertheless, these associations require careful interpretation because eGFR is incorporated into the SCORE2-Diabetes algorithm. Consequently, the correlation between eGFR and SCORE2-Diabetes is partly inherent to the construction of the score and cannot be considered an independent validation of the algorithm. The findings remain clinically informative because they demonstrate that individuals assigned to higher estimated cardiovascular-risk categories also carried a substantially greater burden of renal dysfunction [25].

Unlike eGFR, urinary albumin-to-creatinine ratio did not differ significantly across SCORE2-Diabetes tertiles and was not significantly correlated with the score after correction for multiple testing. Several explanations are possible. Albuminuria has considerable biological variability and may be influenced by marked hyperglycemia, hypertension, heart failure, exercise, infection, and other transient factors. Clinical guidelines therefore recommend confirmation using repeated measurements before persistent albuminuria is diagnosed. As the present database included a single recorded UACR measurement, misclassification and attenuation of associations cannot be excluded [21].

Despite its weak relationship with SCORE2-Diabetes, UACR showed the largest positive standardized coefficient in the elastic-net classification model for established ASCVD. This finding suggests that albuminuria may provide information related to vascular and endothelial injury that is not fully captured by the conventional risk score. However, the coefficient was obtained from a small cross-sectional dataset and should be regarded as exploratory rather than as evidence that UACR is an independently validated predictor in this cohort [26,27].

### 4.3. Cardiovascular-Risk Stratification Using SCORE2-Diabetes

The mean estimated 10-year cardiovascular risk was 34.87%, demonstrating a generally high-risk population. Increasing SCORE2-Diabetes tertiles were accompanied by older age; longer diabetes duration; higher systolic blood pressure; poorer glycemic control; lower eGFR; and a greater prevalence of hypertension, heart failure, and established ASCVD [28].

These results are directionally consistent with the variables and clinical domains incorporated into SCORE2-Diabetes. The algorithm integrates conventional cardiovascular-risk factors with diabetes-specific information, including age at diabetes diagnosis, HbA1c, and eGFR, to estimate the risk of fatal and nonfatal cardiovascular events. Consequently, the strong correlations with age, diabetes duration, HbA1c, systolic blood pressure, and eGFR were expected and should be interpreted as internal descriptive consistency rather than as evidence of external predictive validity [29].

SCORE2-Diabetes is intended for adults aged 40–69 years with T2DM who do not have symptomatic ASCVD or severe target-organ damage [26,27,30]. The full cohort included 49 participants with established ASCVD, 41 with severe target-organ damage according to the available renal and three-site microvascular criteria, and 68 outside of the intended age range; these categories overlapped. Consequently, full-cohort tertiles describe the distribution of recorded score values but must not be used to assign formal treatment categories to ineligible patients.

The new eligibility-restricted sensitivity analysis partly resolves this concern. Among 118 eligible participants, the mean estimated risk was 26.66%, and 72.9% remained in the ≥20% category. Associations with age, HbA1c, diabetes duration, systolic blood pressure, eGFR, FIB-4, and TyG persisted after false-discovery-rate correction. However, because several of these variables are embedded in the SCORE2-Diabetes equation, these findings indicate internal consistency rather than external validation. For clinical classification, established ASCVD or severe target-organ damage should take precedence; SCORE2-Diabetes should be applied only to the remaining eligible population, rather than pooled indiscriminately with high-risk disease states.

### 4.4. Glycemic Control, Inflammation, and Hepatic-Risk Indicators

Both fasting plasma glucose and HbA1c increased significantly across SCORE2-Diabetes tertiles. HbA1c showed a moderate positive correlation with the score, whereas fasting glucose had a weaker association. These findings reflect the importance of sustained glycemic exposure within the broader cardiovascular-risk profile. Nevertheless, the cross-sectional design does not permit determination of whether poorer glycemic control preceded cardiovascular-risk accumulation or resulted from longer and more complex diabetes [31].

CRP concentrations displayed a weak upward trend across SCORE2-Diabetes tertiles but did not differ significantly among groups and were not significantly associated with SCORE2-Diabetes after false-discovery-rate correction. This suggests that systemic inflammation, as measured by a single CRP determination, was not a major discriminator of estimated cardiovascular risk in this sample. The wide distribution of CRP and the presence of several elevated values may have further reduced the stability of the association [32,33].

CRP was weakly associated with body mass index and waist circumference, supporting a relationship between adiposity and low-grade inflammation. However, the negative coefficient observed for CRP in the elastic-net model should not be interpreted as a protective biological effect. In penalized multivariable models, the sign of a coefficient may change when correlated predictors are entered simultaneously, particularly in small datasets with a limited number of outcome events.

FIB-4 was fully reconstructable after treating one biologically impossible platelet value of 0 as missing. Median AST was 23 U/L, median ALT was 22 U/L, median valid platelet count was 239 × 10^3^/µL, and median FIB-4 was 1.26; 5.6% had FIB-4 > 3.25. FIB-4 increased with SCORE2-Diabetes and correlated with age and lower eGFR. Because age is included directly in the FIB-4 equation, this association is partly mathematical, and FIB-4 thresholds may also perform differently in older adults. The score should therefore be regarded as a hepatic-fibrosis risk-screening marker rather than evidence of diagnosed liver fibrosis, and its incremental value requires prospective validation [32,33,34].

### 4.5. Machine-Learning Classification of Established ASCVD

The machine-learning component was designed to classify prevalent ASCVD rather than to reconstruct SCORE2-Diabetes from its constituent variables. SCORE2-Diabetes and the diagnoses defining the composite ASCVD outcome were excluded from the predictor set to limit direct target leakage [35].

After nested tuning, all three algorithms produced ROC AUC values close to 0.67, with strongly overlapping confidence intervals. Random forest and gradient boosting did not outperform penalized logistic regression. This convergence indicates that model complexity did not overcome the restricted information and small event count in the dataset.

The modest performance indicates that prevalent ASCVD could not be classified reliably from the selected cross-sectional biomarkers alone. Smoking status and baseline drug classes were available and are now reported; adding current smoking to the penalized sensitivity model did not clearly improve performance. However, cumulative smoking dose, medication dose and duration, longitudinal treatment exposure, adherence, family history detail, socioeconomic factors, vascular imaging, and temporal biomarker change were not incorporated into the main model.

UACR, diabetes duration, age, eGFR, HbA1c, and FIB-4 had the largest coefficients in the final penalized model, but 49 events for 11 predictors yielded only 4.45 events per candidate predictor. The selected elastic-net setting reached the ridge boundary, consistent with a need for strong shrinkage. Nested cross-validation and penalization reduce optimism but cannot make a small dataset equivalent to an adequately powered development cohort; coefficient ordering and sign should therefore be interpreted as unstable and exploratory.

Nested outer out-of-fold predictions prevented direct reuse of the evaluation observations for preprocessing, hyperparameter tuning, threshold selection, or model fitting. Nevertheless, this remains internal validation. The absence of temporal or external validation, the cross-sectional prevalent-disease outcome, and the wide confidence intervals preclude individual clinical decision-making.

### 4.6. Clinical Implications

The findings support several clinically relevant observations. First, patients with type 2 diabetes frequently present with multiple interacting cardiovascular, renal, and metabolic abnormalities, even when no single biomarker appears severely abnormal. Second, renal evaluation should include both eGFR and UACR because these measures reflect related but noninterchangeable dimensions of kidney and cardiovascular risk. Third, high-risk patients cannot be identified adequately by glycemic measurements alone [36].

For patients with established ASCVD, heart failure, or chronic kidney disease, management should be based on the documented condition rather than exclusively on a calculated risk estimate. The baseline dataset showed substantial use of SGLT2 inhibitors, GLP-1 receptor agonists, renin–angiotensin-system blockers, statins, and antiplatelet/antithrombotic therapy. However, the cross-sectional design and lack of dose, duration, adherence, and longitudinal treatment-change data prevent causal comparison of drug classes or assessment of whether treatment improved outcomes [30].

The present analysis also illustrates that machine learning does not automatically provide superior prediction in relatively small clinical datasets. Model complexity should be proportional to the sample size, number of events, data quality, and intended clinical use. In this cohort, the interpretable penalized model was at least as informative as the more complex algorithms.

### 4.7. Strengths and Limitations

A major strength was the integrated evaluation of demographic, smoking, anthropometric, glycemic, lipid, inflammatory, hepatic-risk, treatment, cardiovascular, and renal variables in the same cohort. Recalculation of FIB-4; explicit SCORE2-Diabetes eligibility analysis; correction for multiple testing; penalization; nested cross-validation; training-only threshold selection; and evaluation through ROC AUC, average precision, Brier score, sensitivity, specificity, and balanced accuracy improved reproducibility and analytical transparency.

Several limitations must be acknowledged. First, the cross-sectional design prevents assessment of temporality, causality, or prediction of incident cardiovascular events. The machine-learning outcome represented prevalent, previously established ASCVD rather than prospectively observed events.

Second, this was a fixed cohort, and no a priori minimum sample-size calculation was performed. Only 49 ASCVD cases were available for 11 candidate predictors (4.45 events per predictor), limiting precision and coefficient stability despite penalization and nested validation.

Third, full-cohort SCORE2-Diabetes tertiles included ineligible participants and therefore remain descriptive. The eligibility-restricted analysis improves formal applicability but was smaller (*n* = 118), and severe target-organ damage could be operationalized only from variables recorded in the database.

Fourth, smoking category and baseline treatment classes were available and have now been reported, but cumulative exposure, medication dose, treatment duration, treatment changes, adherence, detailed family history, socioeconomic status, cardiac imaging, and repeated biomarker measurements were unavailable or incompletely represented.

Fifth, albuminuria and CRP were based on single measurements, and one biologically impossible platelet value reduced valid FIB-4 observations to 231. Finally, the models underwent only internal nested cross-validation and require temporal and external validation in larger, ethnically and geographically diverse cohorts before any clinical implementation.

## 5. Conclusions

Adults with type 2 diabetes in this real-world cohort exhibited a substantial and overlapping burden of cardiovascular, renal, and metabolic abnormalities. Hypertension, dyslipidemia, obesity, elevated TyG index, suboptimal glycemic control, albuminuria, reduced eGFR, heart failure, and established ASCVD were frequent.

In the formal eligibility-restricted subgroup, higher SCORE2-Diabetes values remained associated with older age, longer diabetes duration, higher systolic blood pressure, poorer glycemic control, and lower eGFR. These relationships are partly structural because several variables are included in the algorithm. Patients with established ASCVD or severe target-organ damage should be classified directly by those conditions rather than by SCORE2-Diabetes.

Nested internally validated machine-learning models based on routinely available cardio–renal–metabolic variables showed only modest discrimination of prevalent ASCVD (ROC AUC approximately 0.67) with wide uncertainty. They are exploratory classifiers and are not sufficiently accurate or externally validated for clinical deployment.

These findings support integrated cardio–renal–metabolic assessment while emphasizing transparent eligibility rules, reproducible hepatic-risk calculation, and direct recognition of established disease. Larger prospective multicenter cohorts with longitudinal outcomes, complete treatment exposure and adherence data, and external validation are required before machine-learning results can influence clinical decisions.

## Figures and Tables

**Table 1:** Baseline demographic, clinical, and cardio–renal–metabolic characteristics of the study cohort.

Characteristic	Total Cohort (*N* = 232)
**Demographic characteristics**	
Age, years	62.58 ± 10.10
Female sex	122 (52.6%)
Male sex	110 (47.4%)
Urban residence	119 (51.3%)
Rural residence	113 (48.7%)
Duration of diabetes, years	6.0 (2.0–14.0)
**Anthropometric characteristics**	
Body mass index, kg/m^2^	31.21 ± 5.70
Normal weight	26 (11.2%)
Overweight	79 (34.1%)
Class I obesity	77 (33.2%)
Class II obesity	29 (12.5%)
Class III obesity	21 (9.1%)
Any obesity	127 (54.7%)
Waist circumference, cm	106.13 ± 13.99
**Blood pressure and cardiometabolic comorbidities**	
Systolic blood pressure, mmHg	145.87 ± 20.48
Diastolic blood pressure, mmHg	86.69 ± 11.21
Hypertension	208 (89.7%)
Dyslipidemia	219 (94.4%)
Established atherosclerotic cardiovascular disease	49 (21.1%)
Previous myocardial infarction	23 (9.9%)
Previous coronary revascularization	20 (8.6%)
Previous stroke	14 (6.0%)
Peripheral arterial disease	25 (10.8%)
Heart failure	61 (26.3%)
Atrial fibrillation	18 (7.8%)
**Glycemic profile**	
Fasting plasma glucose, mg/dL	143.5 (118.0–188.5)
HbA1c, %	7.00 (6.25–8.60)
Triglyceride–glucose index	9.15 (8.79–9.71)
Triglyceride–glucose index >9.04	137 (59.1%)
**Renal profile**	
Serum creatinine, mg/dL	0.83 (0.66–1.00)
Estimated glomerular filtration rate, mL/min/1.73 m^2^	82.97 (59.91–94.89)
eGFR ≥90 mL/min/1.73 m^2^	85 (36.6%)
eGFR 60–89 mL/min/1.73 m^2^	88 (37.9%)
eGFR 30–59 mL/min/1.73 m^2^	55 (23.7%)
eGFR <30 mL/min/1.73 m^2^	4 (1.7%)
Urinary albumin-to-creatinine ratio, mg/g	15.87 (11.49–46.91)
Urinary albumin-to-creatinine ratio ≥30 mg/g	80 (34.5%)
Inflammatory and cardiovascular-risk profile	
C-reactive protein, mg/L	3.50 (1.50–6.93)
SCORE2-Diabetes estimated 10-year cardiovascular risk, %	34.87 ± 15.71
**Additional smoking and anthropometric characteristics**	
Current smoker	29 (12.5%)
Former smoker	40 (17.2%)
Never smoker	163 (70.3%)
Waist-to-height ratio	0.636 ± 0.086
Waist-to-height ratio >0.5	225 (97.0%)

Data are presented as mean ± standard deviation, median (interquartile range), or number (percentage), as appropriate. Body mass index categories were derived from the recorded individual BMI values: normal weight, 18.5–24.9 kg/m^2^; overweight, 25.0–29.9 kg/m^2^; class I obesity, 30.0–34.9 kg/m^2^; class II obesity, 35.0–39.9 kg/m^2^; and class III obesity, ≥40.0 kg/m^2^. Established atherosclerotic cardiovascular disease was defined by the presence of at least one of the following: previous myocardial infarction, coronary revascularization, stroke, or peripheral arterial disease. HbA1c was available for 231 participants after one clinically implausible value of 114% was treated as missing. All other variables included in the table were available for the entire cohort. **Abbreviations:** eGFR, estimated glomerular filtration rate; HbA1c, glycated hemoglobin; SCORE2-Diabetes, Systematic Coronary Risk Evaluation 2—Diabetes.

**Table 2:** Biochemical, hepatic-risk, and baseline treatment characteristics of the study cohort.

Variable	Total Cohort (*N* = 232)
Fasting plasma glucose, mg/dL	143.5 (118.0–188.5)
HbA1c, % *	7.00 (6.25–8.60)
Serum creatinine, mg/dL	0.83 (0.66–1.00)
eGFR, mL/min/1.73 m^2^	82.97 (59.91–94.89)
Albumin-to-creatinine ratio, mg/g	15.87 (11.49–46.91)
Albuminuria ≥ 30 mg/g	80 (34.5%)
C-reactive protein, mg/L	3.50 (1.50–6.93)
TyG index	9.15 (8.79–9.71)
TyG index > 9.04	137 (59.1%)
SCORE2-Diabetes, %	34.87 ± 15.71
**Hepatic-risk variables**	
AST, U/L	23 (18–29)
ALT, U/L	22 (15–32)
Platelet count, ×10^3^/µL *	239 (194–285)
FIB-4 *	1.26 (0.96–1.80)
FIB-4 < 1.45 *	146 (63.2%)
FIB-4 1.45–3.25 *	72 (31.2%)
FIB-4 > 3.25 *	13 (5.6%)
**Glucose-lowering therapy**	
Metformin/biguanide	123 (53.0%)
Sulfonylurea	19 (8.2%)
DPP-4 inhibitor	34 (14.7%)
SGLT2 inhibitor	104 (44.8%)
GLP-1 receptor agonist	36 (15.5%)
Basal insulin	73 (31.5%)
Rapid-acting insulin	22 (9.5%)
**Cardiovascular therapy**	
Beta-blocker	125 (53.9%)
Diuretic	115 (49.6%)
ACE inhibitor	111 (47.8%)
Angiotensin-receptor blocker	59 (25.4%)
Antiplatelet/antithrombotic therapy	75 (32.3%)
Statin	113 (48.7%)
Ezetimibe	28 (12.1%)

* HbA1c available for 231 participants after exclusion of one clinically implausible value. * Platelet count and FIB-4 were available for 231 participants after one platelet value of 0 was treated as biologically implausible. Treatment classes overlap; percentages do not sum to 100%.

**Table 3:** Clinical and cardio–renal–metabolic characteristics according to SCORE2-Diabetes risk tertiles.

Characteristic	T1: ≤26.1% (*n* = 78)	T2: >26.1% to <42.2% (*n* = 76)	T3: ≥42.2% (*n* = 78)	*p*-Value
Age, years	53.99 ± 6.34	63.64 ± 8.10	70.14 ± 8.26	<0.001
Female sex	34 (43.6%)	34 (44.7%)	54 (69.2%)	0.001
Diabetes duration, years	2.50 (1.00–6.00)	8.00 (3.75–15.00)	10.00 (4.00–19.00)	<0.001
Body mass index, kg/m^2^	31.92 ± 5.71	31.20 ± 5.64	30.51 ± 5.73	0.306
Waist circumference, cm	106.09 ± 14.48	106.32 ± 13.84	106.00 ± 13.82	0.990
Systolic blood pressure, mmHg	138.81 ± 19.31	144.09 ± 16.80	154.67 ± 21.88	<0.001
Diastolic blood pressure, mmHg	88.62 ± 10.37	85.93 ± 9.31	85.51 ± 13.40	0.174
Hypertension	63 (80.8%)	67 (88.2%)	78 (100.0%)	<0.001
Dyslipidemia	70 (89.7%)	72 (94.7%)	77 (98.7%)	0.051
Established ASCVD	12 (15.4%)	13 (17.1%)	24 (30.8%)	0.036
Heart failure	9 (11.5%)	23 (30.3%)	29 (37.2%)	<0.001
Fasting plasma glucose, mg/dL	129.00 (107.75–157.00)	146.50 (123.50–190.25)	167.50 (123.50–225.75)	<0.001
HbA1c, %	6.40 (6.00–7.07)	7.20 (6.30–8.53)	7.90 (6.90–10.30)	<0.001
TyG index	9.02 (8.73–9.44)	9.14 (8.79–9.84)	9.31 (8.86–9.85)	0.039
Serum creatinine, mg/dL	0.78 (0.65–0.88)	0.83 (0.66–1.01)	0.92 (0.68–1.12)	0.003
eGFR, mL/min/1.73 m^2^	94.15 (81.25–101.34)	80.43 (60.20–92.25)	64.41 (47.83–87.20)	<0.001
Urinary albumin-to-creatinine ratio, mg/g	15.62 (11.73–35.63)	14.29 (10.87–36.64)	22.92 (11.40–89.25)	0.132
C-reactive protein, mg/L	2.90 (1.32–6.57)	3.70 (1.45–5.33)	4.60 (1.90–9.25)	0.086
Waist-to-height ratio	0.623 (0.556–0.685)	0.628 (0.575–0.688)	0.635 (0.593–0.697)	0.428

Data are presented as mean ± standard deviation, median (interquartile range), or number (percentage). One-way analysis of variance was used for normally distributed continuous variables, the Kruskal–Wallis test for non-normally distributed continuous variables, and Pearson’s chi-square test for categorical variables. HbA1c was available for 231 participants because one clinically implausible value was treated as missing. Established ASCVD was defined as a history of myocardial infarction, coronary revascularization, stroke, or peripheral arterial disease. **Abbreviations:** ASCVD, atherosclerotic cardiovascular disease; CRP, C-reactive protein; eGFR, estimated glomerular filtration rate; HbA1c, glycated hemoglobin; IQR, interquartile range; SCORE2-Diabetes, Systematic Coronary Risk Evaluation 2—Diabetes; TyG, triglyceride–glucose.

**Table 4:** Spearman correlations between SCORE2-Diabetes and cardio–renal–metabolic variables.

Variable	*n*	Spearman ρ	Unadjusted *p*-Value	FDR-Adjusted *p*-Value
Age	232	0.736	<0.001	<0.001
HbA1c	231	0.424	<0.001	<0.001
Diabetes duration	232	0.389	<0.001	<0.001
Systolic blood pressure	232	0.359	<0.001	<0.001
Serum urea	232	0.354	<0.001	<0.001
FIB-4	231	0.323	<0.001	<0.001
Fasting plasma glucose	232	0.289	<0.001	<0.001
Serum creatinine	232	0.223	<0.001	0.001
TyG index	232	0.171	0.009	0.017
C-reactive protein	232	0.139	0.035	0.055
Urinary albumin-to-creatinine ratio	232	0.130	0.048	0.070
Total cholesterol	232	0.068	0.300	0.380
LDL cholesterol	232	0.059	0.374	0.445
Waist circumference	232	0.053	0.420	0.469
Triglycerides	232	0.031	0.635	0.670
HDL cholesterol	232	0.002	0.973	0.973
Body mass index	232	−0.080	0.224	0.304
Diastolic blood pressure	232	−0.139	0.035	0.055
Estimated glomerular filtration rate	232	−0.446	<0.001	<0.001

Correlations were calculated using Spearman’s rank correlation coefficient. False discovery rate-adjusted *p*-values were calculated using the Benjamini–Hochberg procedure across the 19 comparisons with SCORE2-Diabetes. HbA1c and FIB-4 were available for 231 participants. One clinically implausible HbA1c value was treated as missing. Correlations with variables included in, or closely related to components of, the SCORE2-Diabetes algorithm should be interpreted as descriptive rather than as evidence of independent predictive validity. Abbreviations: CRP, C-reactive protein; eGFR, estimated glomerular filtration rate; FDR, false discovery rate; FIB-4, Fibrosis-4 Index; HbA1c, glycated hemoglobin; SCORE2-Diabetes, Systematic Coronary Risk Evaluation 2—Diabetes; TyG, triglyceride–glucose.

**Table 5:** Nested cross-validated performance of machine-learning models for the classification of established ASCVD.

Model	ROC AUC (95% CI)	Average Precision (95% CI)	Brier Score	Sensitivity	Specificity	Balanced Accuracy
Elastic-net logistic regression	0.675 (0.582–0.763)	0.368 (0.265–0.523)	0.156	67.3%	63.4%	65.4%
Random forest	0.670 (0.581–0.754)	0.395 (0.272–0.534)	0.156	55.1%	70.5%	62.8%
Gradient boosting	0.674 (0.589–0.752)	0.402 (0.281–0.535)	0.156	40.8%	71.6%	56.2%

Performance estimates were derived from outer out-of-fold predictions in nested stratified five-fold cross-validation. All preprocessing and hyperparameter tuning were performed within training data. Confidence intervals were calculated from 3000 subject-level bootstrap resamples. Sensitivity, specificity, and balanced accuracy used Youden thresholds derived solely from inner out-of-fold predictions in each outer training set. ASCVD prevalence was 21.1%.

**Table 6:** Descriptive Standardized Coefficients From the Final Penalized Logistic-Regression Model.

Predictor	Standardized Coefficient, β	Exponentiated Coefficient
Urinary albumin-to-creatinine ratio	0.227	1.254
Diabetes duration	0.183	1.200
Age	0.179	1.196
Estimated glomerular filtration rate	−0.177	0.838
HbA1c	0.126	1.134
FIB-4	0.123	1.131
C-reactive protein	−0.090	0.914
TyG index	−0.076	0.927
Body mass index	−0.061	0.941
Male sex	0.029	1.029
Systolic blood pressure	−0.019	0.981

Coefficients are from the final model refitted on the complete cohort after cross-validated tuning selected C = 0.03 and l1 ratio = 0. Exponentiated coefficients represent changes per one-standard-deviation increase in continuous predictors. They are provided for descriptive interpretability only and are not independently validated odds ratios or causal effects.

## Data Availability

The original contributions presented in this study are included in this article. Further inquiries can be directed to the first authors.

## Abbreviations

The following abbreviations are used in this manuscript:

Abbreviation	Definition
AUC	Area under the curve
BMI	Body mass index
CI	Confidence interval
CRP	C-reactive protein
DPP-4	Dipeptidyl peptidase-4
eGFR	Estimated glomerular filtration rate
FIB-4	Fibrosis-4 index
GLP-1	Glucagon-like peptide-1
HbA1c	Glycated hemoglobin
HDL	High-density lipoprotein
IQR	Interquartile range
LDL	Low-density lipoprotein
OR	Odds ratio
ROC	Receiver operating characteristic
SD	Standard deviation
SGLT2	Sodium–glucose cotransporter-2
T0	Baseline assessment
T1	Six-month follow-up assessment
T2DM	Type 2 diabetes mellitus
TyG	Triglyceride–glucose index
UACR	Urinary albumin-to-creatinine ratio
